# Concomitant spinal dural arteriovenous fistula and nodular fasciitis in an adolescent: case report

**DOI:** 10.1186/s12887-021-03032-0

**Published:** 2022-01-08

**Authors:** Chan-Lin Chu, Yu-Jen Lu, Tsong-Hai Lee, Shih-Ming Jung, Yu-Cheng Chu, Ho-Fai Wong

**Affiliations:** 1 Department of Neurology, New Taipei Municipal Tucheng Hospital, Chang Gung Memorial Hospital, New Taipei City, Taiwan; 2 College of Medicine, Chang Gung University, Taoyuan, Taiwan; 3 Department of Neurosurgery, Linkou Chang Gung Memorial Hospital, Taoyuan, Taiwan; 4 Department of Neurology, Linkou Chang Gung Memorial Hospital, Taoyuan, Taiwan; 5 Department of Pathology, Linkou Chang-Gung Children Hospital, Chang-Gung Memorial Hospital, Taoyuan, Taiwan; 6 Department of Critical Care, Far-Eastern Hospital, New Taipei City, Taiwan; 7 Division of Neuroradiology, Department of Medical Imaging and Intervention, Linkou Chang-Gung Memorial Hospital, No.5, Fuxing Street, Guishan Township, Taoyuan, Taiwan

**Keywords:** Spinal dural arteriovenous fistula, Pediatric, Embolization, Spinal tumor, Nodular fasciitis, Case report

## Abstract

**Background:**

Spinal dural arteriovenous fistula (SDAVF) usually occurs during the 4^th^ to 6^th^ decades of life, and adolescent SDAVF is rarely reported. SDAVF arising around a tumor is also rare, and reported tumors are mostly schwannoma and lipoma.

**Case presentation:**

We reported a 16-year-old male presented with progressive weakness and numbness of lower limbs for 3 months. A SDAVF was found, which was fed by right radicular arteries from segmental artery at L2 level and drained retrogradely into perimedullary veins. A concomitant spinal extradural nodular fasciitis at right L1/L2 intervertebral foramen was also noted. The SDAVF was completely obliterated by endovascular treatment and the tumor was debulked. The patient recovered well after the procedures.

**Conclusions:**

Our case report suggests SDAVF can occur in adolescent. The concomitant presence with a nodular fasciitis indicates that although it usually arises in subcutaneous tissue but can rarely form on the dura of spine.

## Background

Spinal dural arteriovenous fistula (SDAVF) is a type of arteriovenous shunt on the dural sleeve of spinal cord. It is often regarded as an acquired disease with mean age of diagnosis at age 55-60 years, and young adult SDAVF constituted less than 1% of all SDAVF [1]. The clinical presentation of SDAVF is nonspecific and often misdiagnosed as polyneuropathy, inflammatory myelopathy, degenerative spine disease, spinal muscular atrophy, and medullary tumor, especially in young patient [1]. On rare occasions, SDAVF may be present concomitantly with spinal tumors; the reported tumors include schwannoma and lipoma [2, 3]. We reported a 16-year-old adolescent with a SDAVF arising around a nodular fasciitis (NF) located at the intervertebral foramen of lumbar spine. The SDAVF was embolized and the nodular fasciitis was removed and confirmed by histopathology. The imaging characteristics and differential diagnosis of nodular fasciitis, endovascular treatment of SDAVF, and the relationships between the SDAVF and NF were discussed.

## Case presentation

**Fig. 1:**
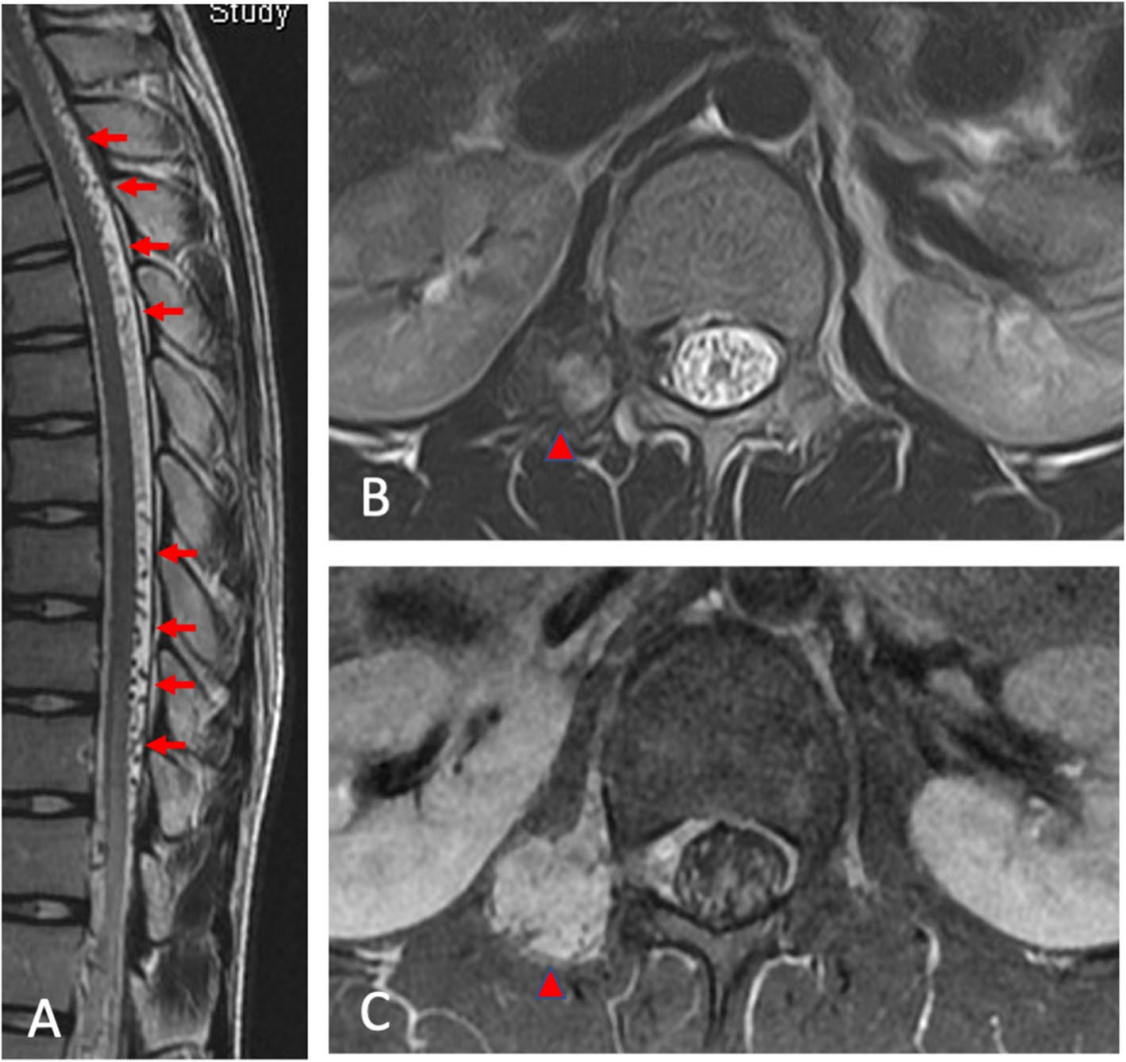
Sagittal spine MR T2-weighted image demonstrated flow voids at perimedullary spaces (A, arrows). Axial views at the level of right L1/L2 intervertebral foramen revealed the spinal dural arteriovenous fistula (SDAVF) arose around a circumscribed tumor which was heterogeneously hyperintense on T2-weighted images (B, arrowhead) and diffusely enhanced on post-contrast T1-weighted images (C, arrowhead)

A 16-year-old male presented with progressive numbness and weakness of lower limbs for 3 months. His development is normal and has no previous disease nor recent trauma history. On admission, he had proximal and distal weakness of lower limbs with muscle power scores of 4 on Medical Research Council Scale, and there was also decreased sensation to light touch and pin-prick below L1 dermatome. He denied incontinence or retention of stool and urine passage. Spinal Magnetic Resonance Imaging (MRI) revealed a longitudinal serpentine flow voids along the perimedullary space from cervical to lumbar spine on T2-weighted images. A circumscribed tumor was also found at right L1/L2 segment, which was heterogeneously hyperintense on T2-weighted image and diffusely enhanced by contrast media (Fig. 1).

Under the impression of spinal arteriovenous fistula, spinal angiography was performed. A SDAVF was found, which was fed by radicular arteries from right L2 segmental artery with a retrograde drainage through an arterialized radicular vein into ventral and dorsal perimedullary veins.

**Fig. 2:**
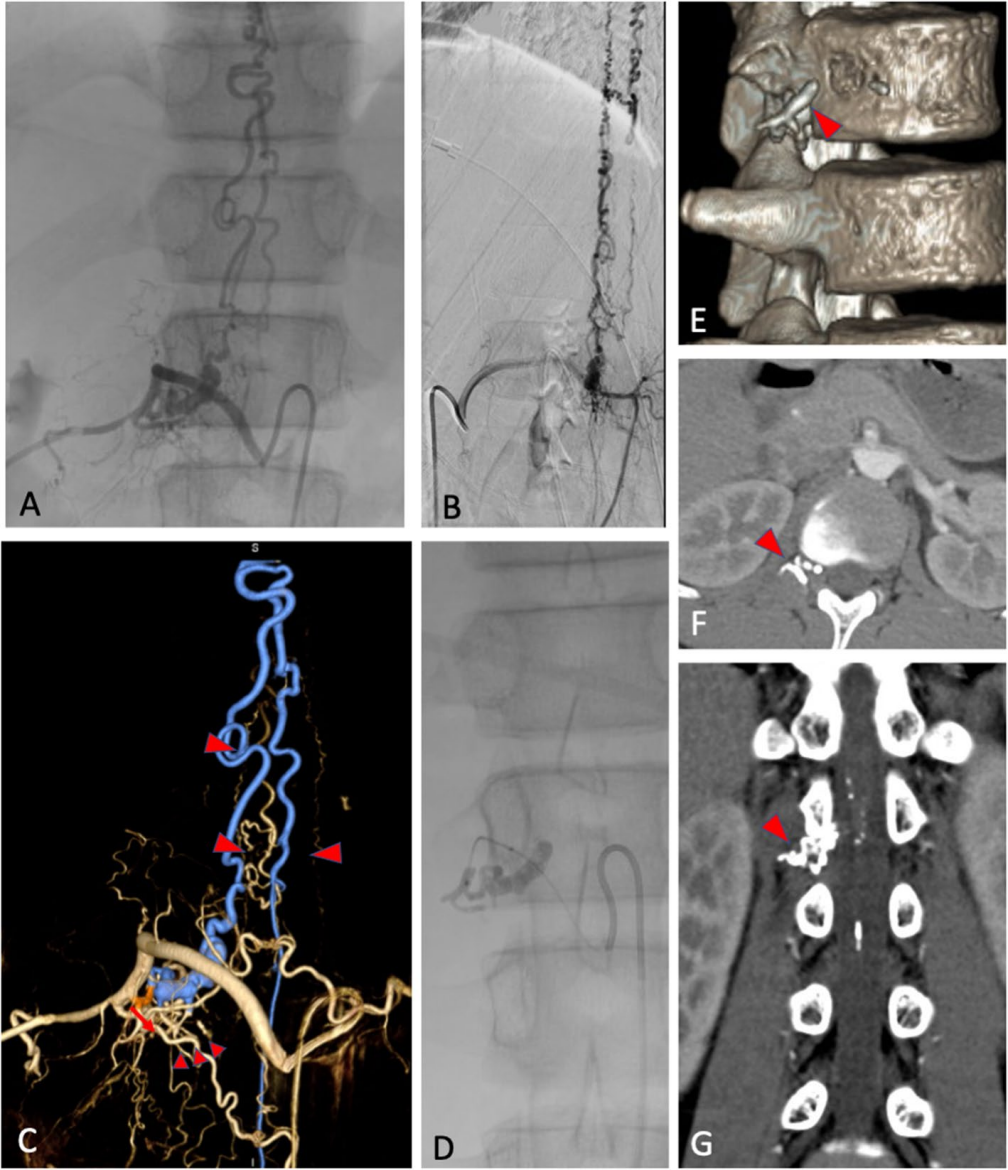
Antero-posterior (**A**) and lateral (**B**) views and three-dimensional reconstruction angiogram (**C**) of the SDAVF from right L2 segmental artery demonstrated the fistula (arrow) was fed by radicular arteries and drained retrogradely through an arterialized radicular vein (small arrowheads) into perimedullary veins (large arrowheads). The n-butyl cyanoacrylate (NBCA) occluded the radicular arteries and proximal draining vein (**D**). 3D reconstruction (**E**), axial (**F**), and axial view (**G**) of the computed tomogram demonstrated the NBCA casting (arrowheads) around the tumor at the right L1/L2 intervertebral foramen

Transarterial embolization was performed under general anesthesia. A guide catheter was placed at the orifice of right L2 segmental artery. A microcatheter was advanced using a micro guidewire into the radicular artery to approach the fistula point. Embolization was performed by using 25% N-butyl cyanoacrylate to occlude the feeder and proximal draining vein. Post-embolization angiography showed total obliteration of the fistula (Fig. 2).

The fistula was found being present around a tumor at right L1/L2 intervertebral foramen. The white, elastic extradural tumor was then surgically removed. The patient recovered uneventfully after the embolization and surgery, and the numbness and weakness disappeared.

**Fig. 3:**
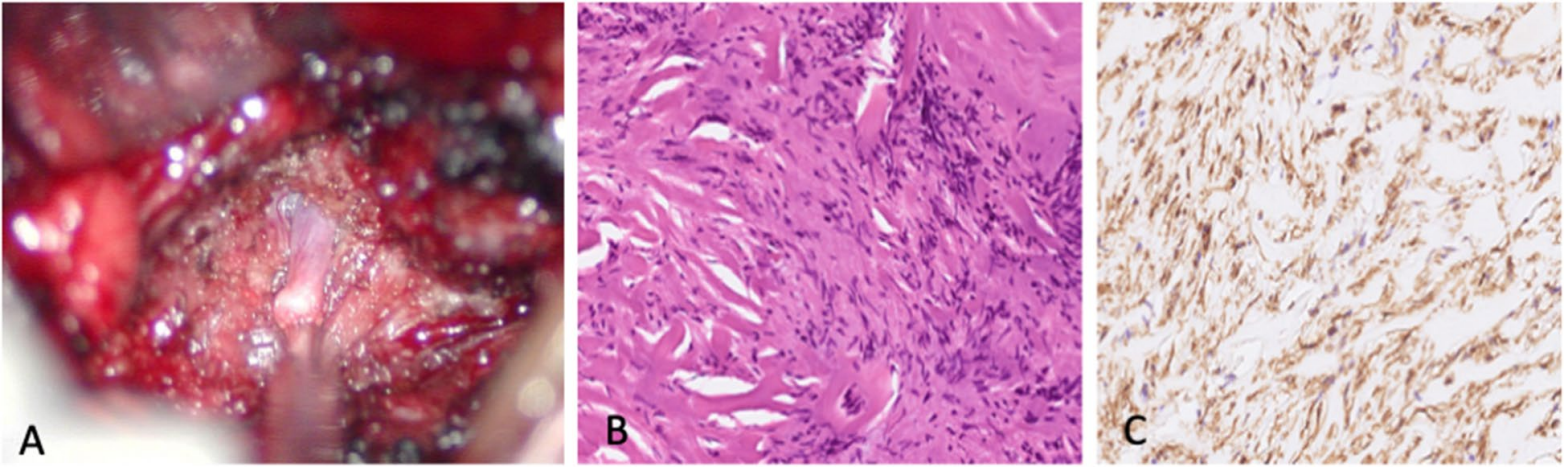
The arteriovenous shunt was identified around the extradural nodular fasciitis (**A**), which was composed of fibrohistiocytic tissue with spindle cells (**B**, H&E) and positive on smooth muscle actin stain (**C**)

Histology examination of the surgical specimens showed fibrohistiocytic tissue and spindle cells with mild nuclear atypia. No mitotic activity and necrosis were found. Immunohistochemical studies showed the tumor was positive for smooth muscle actin and H3K28me3, and was negative for S100, ALK, EMA, beta-catenin, desmin, MUC-4, myogenin, Pan-Trk, and SOX10. The diagnosis was nodular fasciitis (Fig. 3).

## Discussion and conclusions

To our knowledge, the present case is the first reported definite pediatric SDAVF, and also the first SDAVF with concomitant spinal nodular fasciitis. Previously reported tumors concomitant with SDAVF include mainly lipoma and schwannoma [2, 3]. The typical MRI features of SDAVF include serpentine flow voids that represent dilated perimedullary vessels and hyperintense and swollen spinal cord that represents edema on T2-weighted images [4]. Hayward et al. reported a child with “SDAVF” and provided a table listing prior observations of SDAVFs in pediatric populations [5]. However, Pearl and Gailloud [6] suggested the cases reported by Hayward et al. should be labeled as perimedullary AVFs, which is common in pediatric populations, instead of SDAVFs, judging from the angiographic features of the lesion, which consisted of a T11 posterior radiculomedullary feeder and a marked venous pouch at the dorsal side of the spinal cord. Rajadurai et al. [7] also reported a 2-year-old child with “SDAVF” at thoracic spinal cord. However, the presented images also showed a large venous aneurysm in the spinal cord, which is typical of perimedullary AVF and unusual in SDAVF [8]; unfortunately, the authors did not provide enough information to differentiate between the 2 entities. The locations of fistula points are different between SDAVF and perimedullar AVF: In SDAVF, the shunting point is located inside the dura mater close to the spinal nerve root where the arterial blood from a radiculomeningeal artery enters a radicular vein where the latter passes the dura in the lateral epidural space [9]; in perimedullary AVF, these are located on the pial surface of spinal cord at the transition from the spinal artery to the medullary vein [9]. In perimedullary large-volume single-hole fistulas, a large venous pouch is typically present [9].

Nodular fasciitis is a benign, self-limited proliferative myofibroblastic tumor that most commonly occurs in young people [10, 11]. The tumor commonly arises from subcutaneous tissues of extremity, head, and trunk, but can also form in muscle [11], nerve [12], joint [13], vessel [14], and fascia [15]. The reported etiologies include trauma, clonal myofibroblastic tumor, inflammation, and infection. Including our case, only 3 spinal extradural nodular fasciitis have been reported, but one of these is likely to be solitary fibrous tumor instead of nodular fasciitis by histologic finding [16].

The imaging differential diagnosis for spinal extradural nodular fasciitis includes sarcoma, desmoid tumor, neurofibroma, schwannoma, meningioma, fibrous histiocytoma, early myositis ossificans, and malignant lymphoma [17]. Fine needle aspiration cytology can be utilized to assist in differentiating between benign and malignant ones and avoid overly aggressive treatment [18]. The histologic differential diagnosis is fibrohistiocytic tissue with spindle cell tumors and includes neurogenic tumors and fibroblastic/myofibroblastic tumors [15].

SDAVFs usually become symptomatic during the age of 4^th^ to 6^th^ decades and is presumed an acquired disease [1]. Angiogenic factors have been shown to be important in SDAVF formation [3], and it was suggested that the release of angiogenic factors from the lipoma is responsible for fistula formation in cases with concomitant SDAVF and lipoma [3]. Because no prior case of SDAVF with concomitant extradural nodular fasciitis has been reported, it is more reasonable to think that the extradural nodular fasciitis leads to the SDAVF formation, or a common etiologic factor, such as trauma, leads to the occurrence of both [19, 20]. We proposed two putative mechanisms that may lead to fistula formation around the nodular fasciitis. The first one is that angiogenic factors is released from the ischemic tissue secondary to venous hypertension, which is caused by mechanical compression of the venous outlet by the nodular fasciitis. The second one is that nodular fasciitis may disrupt the intradural vessel walls, either by direct invasion or by inducing myofibroblastic change of the vessels through the myofibroblastic “field effect” [14].

When endovascular treatment is considered for SDAVF, it is important to make sure that no spinal cord-suppling arteries are derived from the same pedicle feeding the fistula. It is also important to occlude not only the fistula point but also the most proximal part of the draining vein to prevent intradural collaterals filling the fistula and subsequently recurrence [9]. When the proximal draining vein cannot be occluded or the feeder also supplies the anterior spinal artery, posterior spinal artery, or a radiculomedullary artery, surgery remains the treatment of choice [21].

We report a rare case of adolescent SDAVF with concomitant extradural nodular fasciitis. The typical imaging features on spinal cord MRI include serpentine flow voids that represent dilated perimedullary vessels and hyperintense and swollen spinal cord that represents edema on T2-weighted images. Endovascular treatment of the fistula can be safely performed when the pedicle of the feeder does not supply the spinal cord. Nodular fasciitis is a benign neoplasm that may remit spontaneously. Fine needle aspiration cytology could help to differentiate between benign and malignant neoplasms to avoid overly aggressive treatment.

## Data Availability

The patient data is available on request from the corresponding author after obtaining patient’s consent.

## Abbreviations

AVF: arteriovenous fistula
H & E: Haematoxylin and eosin
SDAVFs: Spinal dural arteriovenous fistula
MRI: Magnetic Resonance Imaging
